# Elaboration of an organic beverage based on grape juice with positive nutritional properties

**DOI:** 10.1002/fsn3.2795

**Published:** 2022-03-11

**Authors:** Yasmina Bendaali, Cristian Vaquero, Carmen González, Antonio Morata

**Affiliations:** ^1^ 16771 Department of Chemistry and Food Technology ETSIAAB Universidad Politécnica de Madrid Madrid Spain

**Keywords:** anthocyanins, color, grape juice, isotonic drink, natural beverage, organic

## Abstract

The present study aimed to develop a natural beverage with interesting phytochemical composition and biological activity based on grape juice without added sugars or artificial additives. Two groups of blends were made by diluting concentrate grape juice with a sugar content of 65 °Brix with two different mineral waters (BA: Bezoya with low mineralization; BB: Solan de Cabras with high mineralization). Lemon juice was used for pH correction, and mixtures of extractions of hop with tea and hop with mint were used to increase aroma. Samples were stored under refrigeration (4°C) then subjected to physicochemical and sensory analysis. The results demonstrated that malvidin‐3‐O‐glucoside pigment was the predominant pigment with a concentration ranging from 75.71 ± 12.49 to 84.87 ± 1.70 mg/L. The levels of sugars ranged from 79.90 ± 1.37 to 82.37 ± 0.55 g/L and total soluble solids were between 5.47 ± 0.12 and 5.77 ± 0.06 °Brix. Total acids presented a significant difference, ranging from 1.40 ± 0.00 to 1.43 ± 0.06 g/L in BA samples and from 1.10 ± 0.10 to 1.20 ± 0.00 g/L in BB samples. For 20 days, the color increased in all beverages. However, BA drinks presented higher acidity and higher red color intensity than BB drinks, so the type of water and pH influenced the color of beverages. The sensory evaluation showed that the beverage made with low mineral water and flavored with a mixture of hop with tea was preferred.

## Graphical abstract

Healthier beverage than grape juice. Low sugar content and good polyphenols balance. Good sensory profile with refreshing acidity and attractive color.

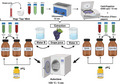

## 1 INTRODUCTION

Currently, the food market is experiencing a great development in the production of natural and functional foods due to the increasing health awareness of consumers and the adoption of healthy eating habits (Chew et al., 2020). The request for organic foods has increased owing to the belief that they might have more benefits than conventional products (Dyab et al., 2015). Accordingly, it has become important to produce new drinks from fruits juices as a source of nutrients and bioactive compounds (Gironés‐Vilaplana et al., 2016a). Consumption of fruits and vegetables is recommended to prevent disease, especially red fruits such as strawberries, cherries, grapes, and pomegranates, which are characterized by their bioactive component (Gardeli et al., 2019). Fruit‐ and vegetable‐based beverages include a variety of polyphenols, oligosaccharides, fibers, and nitrates, which may produce antioxidant, antimicrobial, and antiviral effects (Butu & Rodino, 2019). Nowadays, researchers are interested in the production of foods with lower sugar or alternative sources of sweeteners (Moldovan & David, 2020). Fruit juices could replace sugar sweeteners and contribute to preventing pathologies associated with their consumption (Agulló et al., 2021), such as obesity, diabetes, and cardiovascular disease (Styburski et al., 2020). In addition, among challenges facing the food industry is the expansion in the creation of new products and persuasive consumers to buy them. In this case, it is essential to recognize the needs and the consumer’s demands while developing new and innovative products (Świtalski & Rybowska, 2021). To respond to consumer demand related to the consumption of natural and healthy products, several studies focused on the development of new and healthy beverages based on fruit juice (Bhalerao et al., 2020; Gironés‐Vilaplana et al., 2012, 2013, 2016b; González‐Molina et al., 2012; Shams Najafabadi et al., 2021; Tiencheu et al., 2021). During physical exercise, oxidative stress is induced because of an imbalance between the production of reactive oxygen species and antioxidant capacity in the body, leading to an increase in inflammatory markers, muscle damage, and gastrointestinal dysfunction (AbuMoh’d, 2020; Elejalde et al., 2021; Martins et al., 2020). Grape and its derivatives could be beneficial facing oxidative damage because of the presence of phenolic compounds (De Oliveira et al., 2021). Its carbohydrate content is necessary for glycogen deposition and improvement of practice during long‐term exercise (Martins et al., 2020). Concentrated grape juice is a product obtained by physical methods for removing water and increasing the content of soluble solids present in the respective total juice by at least 50%. By diluting the concentration or dried juice to the initial concentration based on °Brix as reconstitution parameter, reconstituted grape juice is obtained (Dutra et al., 2021). Grape juices consist of water (81%–86%) and a high concentration of sugars (glucose and fructose), with high acidity owing to the existence of organic acids that balance the sweet and sour tastes. They present small amounts of minerals, vitamins, and other phenolic aromatic compounds that provide sensory characteristics of grape juices (color, taste, and flavor) (Cosme et al., 2018; Dutra et al., 2021; García‐Martínez et al., 2021). Among the phenolic compounds found in grape juice are flavonols (kaempferol, quercetin, and myricetin), flavanols (catechin, epicatechin, and procyanidins), anthocyanins (malvidin, cyanidin, delphinidin, petunidin, peonidin, and pelargonidin), phenolic acids, and the stilbene resveratrol (Burin et al., 2010; Lima et al., 2014; Nadeem et al., 2018; O’Byrne et al., 2002; Xia et al., 2010). Consumption of grape juice has positive health advantages due to effective antioxidant, anticarcinogenic, antibacterial, antidiabetic, antiaging, and anti‐inflammatory activities as well as cardioprotective, hepatoprotective, and neuroprotective effects (Nadeem et al., 2018; Wu et al., 2021), which develop endothelial function, increasing the antioxidant capacity of serum and low‐density lipoproteins, minimizing native plasma protein oxidation, and reducing platelet aggregation (Burin et al., 2010; Dávalos et al., 2005). Moreover, studies have shown the beneficial effects of resveratrol and quercetin in the treatment of cancer and cardiovascular diseases (García‐Martínez et al., 2021).

The new drink prepared from grape juice is a natural product in which the use of artificial colorants and flavorings was avoided. No sugars are added, only those of the fruit juice and with great nutritional value and biological activity. Lemon juice is added to the beverage to reduce the pH and provide a pleasant flavor. Lemon is one of the citrus fruits that are characterized by their content of flavanones, vitamin C, minerals, and citric acid, which provide nutritional value in a beverage (Agulló et al., 2021; Gironés‐Vilaplana et al., 2013; González‐Molina et al., 2012). Anthocyanins have a high potential for utilization as natural colorants to replace synthetic dyes in food systems owing to their attractive colors, water solubility, and health benefits (Brenes et al., 2005; Gérar et al., 2019; Tan et al., 2021). Their stability depends on the pH, lack of vitamin C, high concentration of sugar (Cosme et al., 2018), oxygen, light, temperature presence of ascorbic acid, and metal ions (Moldovan & David, 2020; Vidana Gamage et al., 2022).

Among challenges for academic and industrial investigation is the production of natural flavors, which determine the sensory characteristics of beverages and other food products because of the growing preference of consumers for sustainable and natural products (Vilela et al., 2019). Since the prepared drink is an organic product without artificial ingredients, natural flavors extracted from spices and herbs (hop, tea, and mint) are used to enhance the sensory properties and increase the aroma of the beverage.

## 2 MATERIALS AND METHOD

### 2.1 Raw materials

Concentrated red grape juice was used with a sugar content of 65 °Brix, pH 3.5, and SO_2_ <40 ppm (Vinos y Bodegas). Dilutions were performed with mineral waters with different mineralization: Bezoya (Calidad Pascual) and Solan de Cabras.

Table 1 describes the composition of both waters (taken from the label of water bottles). pH correction and flavoring to correct acidity was done with pasteurized squeezed lemon juice (Mercadona), with 40 mg/L of C vitamin and 10 mg/L of sodium. The aroma was improved by infusion extractions from organic red tea (Cafetearte), organic dried mint (Soria Natural), and hop (Summit).

**TABLE 1:** Composition of waters used in the preparation of drinks

Composition	Bezoya (mg/L)	Solan de Cabras (mg/L)
Dry residue (180°C)	28	278
Bicarbonate	21	284
Chlorides	0.60	8.3
Calcium	5.26	60
Magnesium	0.91	26.7
Sodium	1.36	4.8
Potassium	–	1
Silica	9.15	7.5
Nitrates	2.8	–
Sulfates	–	21.8

### 2.2 Extraction

**FIGURE 1:**
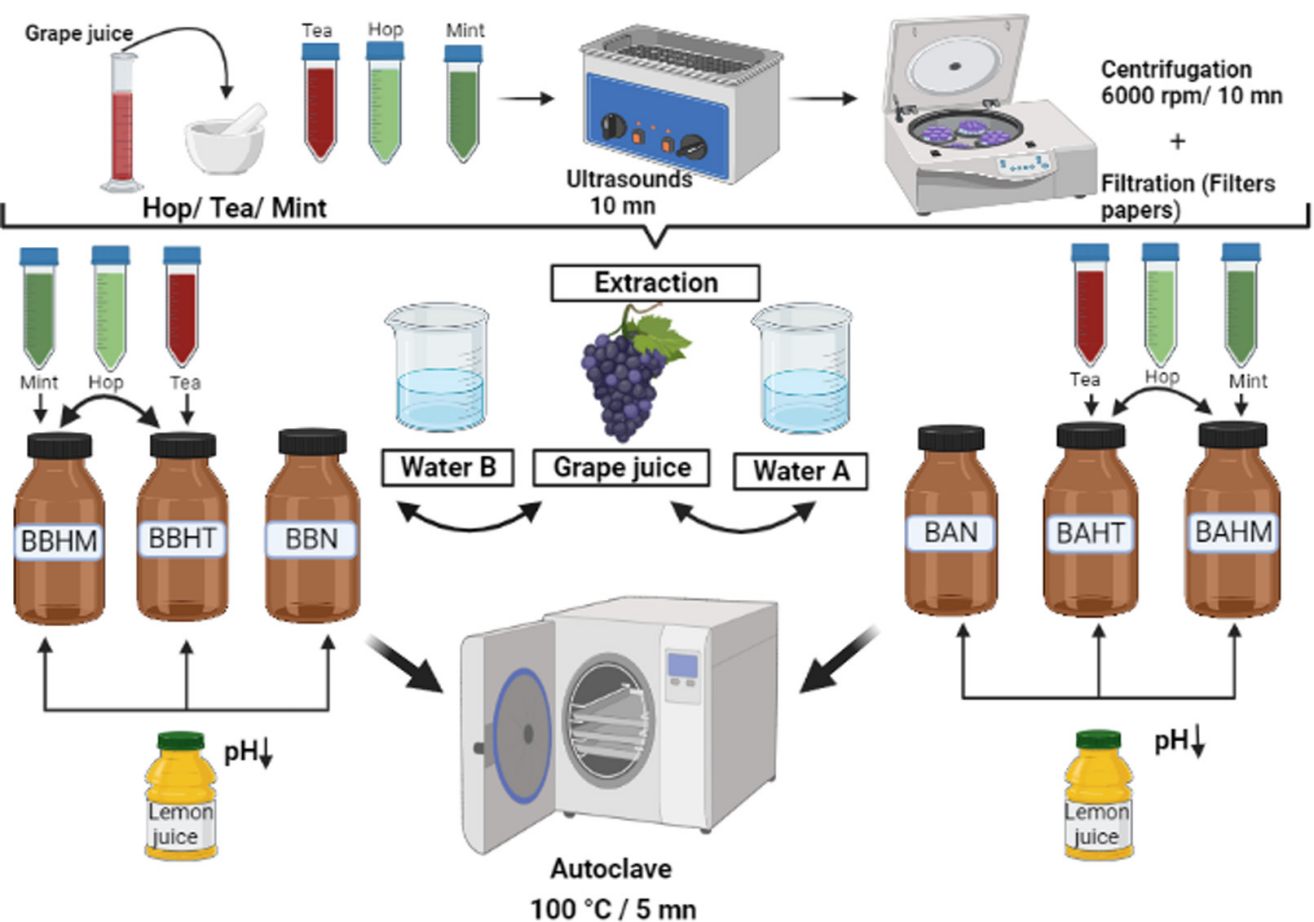
Schematic of flavor extraction and beverage preparation

To obtain the extracts, 4 g of each herb or spice (hop, tea, and mint) were weighed, crushed in the mortar, and mixed with 30 ml of diluted grape juice (Figure 1). The extraction of the aromas and flavors from the three mixtures was performed using ultrasounds (3300EP SONICA) for 10 min followed by centrifugation (Eppendorf, 5430 R) at 6000 rpm at 20°C for 10 min. Finally, the extracts were filtered using filter papers and kept refrigerated at 4°C until being added to the beverages.

### 2.3 Beverage preparation

For the preparation of the beverages, preliminary experiments were carried out, aimed to obtain a product with a suitable sensory profile, in terms of acidity, color, and flavors. Several drinks were prepared with the two types of water previously described (different mineralization) and flavored with different herbs and spices (cardamom, hop, fresh mint, dried mint, black tea, red tea, and green tea). Based on sensory analysis, the appropriate ingredients identified for the final formulation of a natural drink flavored with a mixture of hop‐–tea and hop–mint.

The natural drink was formulated by diluting grape juice (47 ml) with water (453 ml) to obtain 50 g/L of sugar. Using a bottle of 500 ml for each sample (Table 2), two groups of beverages were prepared (Figure 1), the first group "A" using natural mineral water with low mineralization (Bezoya) and the second group "B" using natural mineral water with high mineralization (Solan de Cabras). Six types of drink were prepared: four flavored beverages and two beverages as controls without added flavors. To adjust the acidity, 7 ml of lemon juice was added to each drink. Then, beverages were flavored with a mixture of hop with red tea and hop with mint. Triplicate solutions were prepared for each experiment and all analytical measurements were performed in triplicate.

**TABLE 2:** Drinks formulation: composition and nomenclature

Group	Samples	Water (ml)	Grape juice (ml)	Lemon juice (ml)	Hops (ml)	Tea (ml)	Mint (ml)
A	BAN	453	47	7	–	–	–
	BAHT	453	47	7	2	1	–
	BAHM	453	47	7	2	–	0.5
B	BBN	453	47	7	–	–	–
	BBHT	453	47	7	2	1	–
	BBHM	453	47	7	2	–	0.5

Samples were labeled as follows: BAN (control, beverage with water A without flavoring), BAHT (beverage with water A flavored with hop and red tea), BAHM (beverage with water A flavored with hop and mint), BBN (control, beverage with water B without flavoring), BBHT (beverage with water B flavored with hop and red tea), and BBHM (beverage with water B flavored with hop and mint). The 18 bottles of drinks were thermally treated by autoclave at 100°C for 5 min. Later, they were kept under refrigeration at 4°C until chemical and sensory analysis.

### 2.4 Physicochemical analysis

The Crison brand pH meter GLP 21 model was used for the pH measurements of each sample for 20 days. Sugar concentration (glucose and fructose), total soluble solids (TSS), total acidity, organic acids, and other parameters were identified with OenoFoss^™^ equipment (FOSS Iberia) using Fourier transform infrared spectroscopy.

### 2.5 Color parameters analyzed by UV‐visible spectrophotometry

Since the drink was prepared with grape juice, the same wavelengths (280, 420, 520, and 620 nm) were selected for the measurement. For 20 days, the absorbance was determined using an Agilent 8453 spectrophotometer (Agilent Technologies S.L.) and a 1 mm optical path glass cuvette. The color intensity (CI), the amount of color present in the juices (CI), was obtained by the sum of absorbances at 420, 520, and 620 nm. The tonality (T) was calculated by the quotient between the absorbance values at 420 and 520 nm (Burin et al., 2010). Total phenolic content was the absorbance at 280 nm (Milella et al., 2019).

### 2.6 Determination of anthocyanins

Anthocyanin determination was according to Escott et al. (2017). The anthocyanins were identified and quantified with a series 1200 high‐performance liquid chromatograph (HPLC), equipped with a diode array detector. Twenty‐microliter samples of previously filtered 0.45 µm membrane were injected into the HPLC apparatus. Gradients of solvents A (water/formic acid, 95:5 v/v) and B (methanol/formic acid, 95:5 v/v) were used in a reverse‐phase Poroshell 120 C18 column (Phenomenex) (50 × 4.6 mm; particle size 2.7 µm) as follows: 0–2 min, 15% B (working flow 0.8 ml/min); 2–10 min, 15%–50% B linear; 10–12 min, 50% B; 12–13 min, 50%–15% B linear; and 13–15 min, reequilibration. Detection was performed by scanning in the 400–600 nm range. Quantification was performed by comparison against an external standard at 525 nm and expressed as milligram per liter of malvidin‐3‐O‐glucoside (Extrasynthese) (*r* ^2^ = 0.9999). Anthocyanins were identified by their retention time and by comparing their UV‐visible maximum absorbance. The detection limit was 0.1 mg/L.

### 2.7 Sensory analysis

The sensory evaluation test was carried out in the tasting room of the Department of Chemistry and Food Technology of the Universidad Politécnica de Madrid. The test was performed with eight participants from both genders who were students and teachers aged between 22 and 60 years. Six glasses of beverages prepared were placed on each participant's table at 12 ± 2°C with another glass of water. All the sensory analysis parameters were rated on a scale of 1 (low perception) to 5 (high perception). The attributes evaluated were CI, tonality, turbidity, aromatic intensity, aromatic quality, herbaceous, floral, fruity, reduced, rusty, body, bitterness, sweetness, and acidity.

### 2.8 Statistical analysis

Statgraphics Centurion 18 software V.18.1.06 (Graphics Software Systems) was used to calculate means, standard deviation, and analysis of variance (ANOVA). One‐way ANOVA between groups was performed with the least significant differences. Significance was set at *p* < .05 for the ANOVA matrix. A principal component analysis (PCA) was carried out on the color and anthocyanins parameters using Addinsoft (2021) and XLSTAT statistical and data analysis solution, New York, USA.

## 3 RESULTS

### 3.1 Evolution in pH and color

**FIGURE 2:**
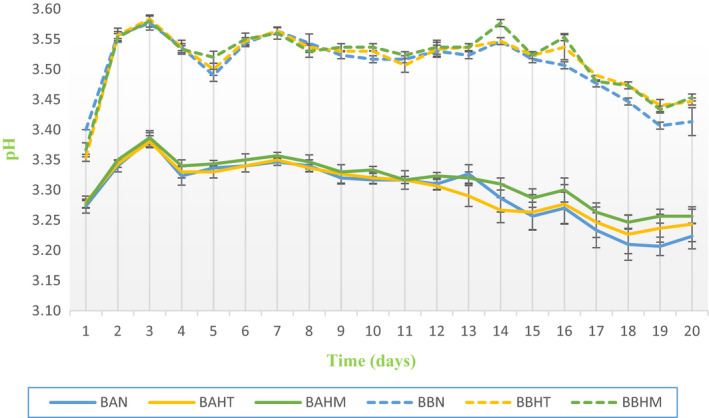
Change in pH values in control beverages and flavored samples for 20 days

Analysis of pH values of beverages (Figure 2) stored under refrigeration at 4°C for 20 days shows a significant difference between the samples (*p* < .05) at the beginning, during, and at the end of the storage period. In BA samples, pH values slightly decreased from 3.27 ± 0.01, 3.28 ± 0.01, and 3.28 ± 0.01, to 3.22 ± 0.02, 3.24 ± 0.03, and 3.26 ± 0.01 in BAN, BAHT, and BAHM, respectively. The BB samples showed a slight increase in pH value, changing from 3.40 ± 0.00, 3.35 ± 0.01, and 3.37 ± 0.01 to 3.41 ± 0.02, 3.45 ± 0.01, and 3.45 ± 0.01 in BBN, BBHT, and BBHM, respectively. All the values were within an acceptable range. They are in line with those found by Galvão et al. (2020) in an isotonic drink enriched with Cajuína, which ranged from 3.58 ± 0.03 to 2.9 ± 0.02 during the storage period and with those pH values found by Gironés‐Vilaplana et al. (2013) of six commercial isotonic beverages, which ranged from 2.64 ± 0.00 to 3.83 ± 0.01.

**FIGURE 3:**
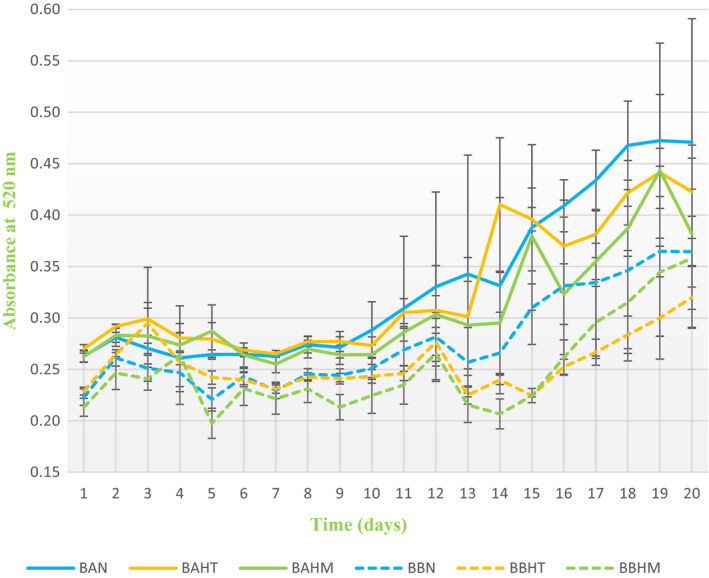
Changes in the absorbance of controls and flavored samples for 20 days at 520 nm

Results in Table 3 demonstrate CI, total phenolic content, and tonality of the two groups of beverages on the first and last day of period storage. The CI values increased in all beverages during the storage period and were higher in BA samples ranging from 0.43 ± 0.00 to 0.51 ± 0.01 on the first day, and from 0.75 ± 0.11 to 1.11 ± 0.36 on the last day. Total phenolic content was between 2.33 ± 0.04 and 2.45 ± 0.02 on the first day, and between 2.43 ± 0.02 and 2.66 ± 0.12 on the last day with significant difference between samples (*p* < .05). For the tonality, values obtained ranged from 0.66 ± 0.01 to 0.77 ± 0.04 on the first day, and from 0.83 ± 0.11 to 0.90 ± 0.07 with no significant difference on the last day. Regarding the absorbance at 520 nm, the length at which anthocyanins absorb, Figure 3 shows an increase in absorbance at 520 nm in all beverages during the storage period. However, the influence of the type of water and acidity on the absorbance of anthocyanins was observed. Drinks with lower pH values and a lower degree of mineralization (BA) presented higher absorbance values than drinks with higher pH values and a higher degree of mineralization (BB).

**TABLE 3:** Color characterization of beverages on the first and last day of the storage period

Parameters	BAN	BAHT	BAHM	BBN	BBHT	BBHM
Total phenolic content (A280 nm)	Day 1 2.36 ± 0.05^ab^	Day 1 2.36 ± 0.05^ab^	Day 1 2.40 ± 0.00^bc^	Day 1 2.33 ± 0.04ª	Day 1 2.36 ± 0.04^ab^	Day 1 2.45 ± 0.02^c^
	Day 20 2.60 ± 0.10^ab^	Day 20 2.66 ± 0.12^b^	Day 20 2.48 ± 0.10^a^	Day 20 2.43 ± 0.02^a^	Day 20 2.47 ± 0.06^a^	Day 20 2.56 ± 0.12^ab^
Color intensity (A420 + A520 + A620)	Day 1 0.49 ± 0.01^c^	Day 1 0.51 ± 0.01^d^	Day 1 0.50 ± 0.01^cd^	Day 1 0.43 ± 0.02^a^	Day 1 0.45 ± 0.01^b^	Day 1 0.43 ± 0.00^a^
	Day 20 1.11 ± 0.36ª	Day 20 0.99 ± 0.16^a^	Day 20 0.87 ± 0.24^a^	Day 20 0.85 ± 0.11^a^	Day 20 0.75 ± 0.11ª	Day 2 0 0.86 ± 0.17ª
Tonality (A420/A520)	Day 1 0.66 ± 0.01ª	Day 1 0.68 ± 0.01^ab^	Day 1 0.68 ± 0.01^ab^	Day 1 0.70 ± 0.00^bc^	Day 1 0.72 ± 0.01^c^	Day 1 0.77 ± 0.04^d^
	Day 20 0.90 ± 0.07ª	Day 20 0.90 ± 0.07ª	Day 20 0.83 ± 0.11ª	Day 20 0.84 ± 0.05ª	Day 20 0.86 ± 0.06ª	Day 20 0.90 ± 0.05ª

Note

Values are means ± *SD* (*n* = 3). A different letter for the same parameter means significant differences (*p* < .05).

### 3.2 Physicochemical analysis

The results of the physicochemical analysis using the FOSS analyzer are presented in Table 4. Samples present a significant difference between the two groups except for malic acid, ammonia, and density. It observed that the samples made with weak mineral water and presented a lower pH were the ones that contained the highest level of sugar content (glucose/fructose) and total acids. The levels of sugars in the beverages ranged from 79.90 ± 1.37 to 82.37 ± 0.55 g/L. TSS were between 5.47 ± 0.12 and 5.77 ± 0.06 °Brix. These results of TSS are close to those of commercial isotonic drinks evaluated by Gironés‐Vilaplana et al. (2013). Total acids present a significant difference between BA samples and BB samples, ranging from 1.40 ± 0.00 to 1.43 ± 0.06 g/L in BA samples and from 1.10 ± 0.10 to 1.20 ± 0.00 g/L in BB samples. Alpha amino acids ranged from 91.53 ± 2.75 to 98.53 ± 1.80 mg/L and were higher in BB samples.

**TABLE 4:** General parameters in beverages measured by FTIR

Parameters	BAN	BAHT	BAHM	BBN	BBHT	BBHM
Glucose/fructose (g/L)	82.07 ± 1.27^bc^	82.37 ± 0.55^c^	81.03 ± 0.78^abc^	79.90 ± 1.37^a^	80.57 ± 0.81^ab^	80.67 ± 0.70^abc^
Total soluble solids (°Brix)	5.63 ± 0.12^bc^	5.77 ± 0.06^c^	5.53 ± 0.12^ab^	5.47 ± 0.12^a^	5.63 ± 0.06^bc^	5.63 ± 0.06^bc^
Total acid (g/L)	1.40 ± 0.00^c^	1.43 ± 0.06^c^	1.43 ± 0.06^c^	1.10 ± 0.10^a^	1.20 ± 0.00^b^	1.20 ± 0.00^b^
Malic acid (g/L)	1.57 ± 0.12^a^	1.60 ± 0.10^a^	1.53 ± 0.06^a^	1.60 ± 0.17^a^	1.57 ± 0.06^a^	1.60 ± 0.00^a^
Alpha amino acids (mg/L)	91.53 ± 2.75^a^	95.80 ± 1.82^ab^	91.63 ± 1.19^a^	91.77 ± 4.48^a^	98.53 ± 1.80^b^	97.63 ± 4.05^b^
Ammonia (mg/L)	28.73 ± 9.00^a^	39.33 ± 4.31^a^	38.30 ± 3.82^a^	34.17 ± 30.73^a^	42.40 ± 8.23^a^	45.33 ± 2.71^a^
Density (g/ml)	1.03 ± 0.00^a^	1.03 ± 0.00^a^	1.03 ± 0.00^a^	1.03 ± 0.00^a^	1.03 ± 0.00^a^	1.03 ± 0.00^a^

Note

Values are means with standard deviations, *n* = 3. Values with the same letter in the same parameter are not significantly different (*p* < .05).

### 3.3 Anthocyanins

The anthocyanin profile of the beverages elaborated was studied by HPLC. The results in Table 5 demonstrated the predominance of malvidin‐3‐O‐glucoside with a concentration between 79.46 ± 8.06 and 84.87 ± 1.70 mg/L in BA beverages and from 75.71 ± 12.49 to 84.24 ± 1.40 mg/L in BB beverages, followed by peonidin‐3‐glucoside with a concentration ranging from 41.39 ± 3.27 to 43.70 ± 1.02 mg/L and from 38.47 ± 5.80 to 43.01 ± 0.76 mg/L in BA and BB, respectively. Our results accord with those obtained in the grape juice by Tiwari et al. (2009), which identified the same three major anthocyanins: cyanidin‐3‐O‐glucoside, delphinidin‐3‐O‐glucoside, and malvidin‐3‐O‐glucoside. Another study by Dutra et al. (2021) reported the presence of malvidin 3‐glucoside, delphinidin 3‐glucoside, peonidin 3‐glucoside, and cyanidin 3‐glucoside in whole grape juice (Gironés‐vilaplana et al., 2012) and also obtained the five similar anthocyanins (delphinidin, cyanidin, petunidin, peonidin, and malvidin) in grape concentrate. Our results show the presence of a concentration between 12.78 ± 2.59 and 14.85 ± 0.56 mg/L of malvidin‐3‐(6”‐p‐coumaroylglucoside) and a low concentration of vitisins (A and B).

**TABLE 5:** Total anthocyanins divided into six beverages

Anthocyanins (mg/L)	BAN	BAHT	BAHM	BBN	BBHT	BBHM
Delphinidin‐3‐O‐glucoside	24.42 ± 0.50^a^	21.53 ± 4.16^a^	22.72 ± 1.30^a^	24.20 ± 0.26^a^	18.80 ± 7.70^a^	20.82 ± 4.33^a^
Cyanidin‐3‐O‐glucoside	10.55 ± 0.20^a^	10.03 ± 0.72^a^	9.96 ± 0.37^a^	10.34 ± 0.14^a^	9.42 ± 1.22^a^	9.38 ± 1.21^a^
Petunidin‐3‐O‐glucoside	20.33 ± 0.41^a^	18.52 ± 2.52^a^	19.25 ± 1.04^a^	20.24 ± 0.26^a^	16.89 ± 4.83^a^	17.74 ± 3.14^a^
Peonidin‐3‐O‐gucoside	43.70 ± 1.02^a^	41.39 ± 3.27^a^	41.67 ± 1.50^a^	43.01 ± 0.76^a^	39.04 ± 5.59^a^	38.47 ± 5.80^a^
Malvidin‐3‐O‐glucoside	84.87 ± 1.70^a^	79.46 ± 8.06^a^	81.09 ± 3.03^a^	84.24 ± 1.40^a^	75.71 ± 12.49^a^	75.77 ± 12.04^a^
Vitisin B	3.65 ± 0.03^c^	3.60 ± 0.03^b^	3.60 ± 0.03^ab^	3.58 ± 0.02^ab^	3.56 ± 0.01^ab^	3.56 ± 0.02^a^
Vitisin A	5.91 ± 0.19^b^	5.68 ± 0.10^ab^	5.63 ± 0.18^ab^	5.68 ± 0.04^ab^	5.33 ± 0.36^a^	5.43 ± 0.28^a^
Cyanidin‐3‐(6″‐acetylglucoside)	3.58 ± 0.04^b^	3.53 ± 0.04^ab^	3.48 ± 0.05^ab^	3.50 ± 0.02^ab^	3.44 ± 0.12^ab^	3.42 ± 0.14^a^
Petunidin‐3‐(6″‐acetylglucoside)	4.03 ± 0.01^a^	3.96 ± 0.10^a^	3.97 ± 0.07^a^	3.99 ± 0.02^a^	3.84 ± 0.26^a^	3.89 ± 0.14^a^
Malvidin‐3‐(6″‐acetylglucoside)	12.05 ± 0.21^a^	11.13 ± 1.15^a^	11.56 ± 0.45^a^	11.73 ± 0.24^a^	10.32 ± 2.24^a^	10.69 ± 1.40^a^
Cyanidin‐3‐(6″‐p‐coumaroyglucoside)	3.49 ± 0.02^a^	3.42 ± 0.06^a^	3.45 ± 0.04^a^	3.46 ± 0.02^a^	3.38 ± 0.13^a^	3.36 ± 0.13^a^
Petunidin‐3‐(6″‐p‐coumaroyglucoside)	4.21 ± 0.05^a^	4.01 ± 0.19^a^	4.12 ± 0.12^a^	4.15 ± 0.05^a^	3.91 ± 0.42^a^	3.93 ± 0.31^a^
Malvidin‐3‐(6″‐p‐coumaroyglucoside)	14.85 ± 0.56^a^	13.55 ± 1.33^a^	14.36 ± 0.99^a^	14.32 ± 0.58^a^	13.18 ± 2.87^a^	12.78 ± 2.59^a^

Note

Values are means ± SD (*n* = 3). Different letters for the same category of anthocyanins mean significant differences (*p* < .05).

### 3.4 Sensory analysis

The results of the sensory analysis on sample preference are presented in Figure 6. Flavored drinks were compared sensorially with the control drink in both groups. In general, in cases of an appreciable difference between flavored drinks and control drinks, tasters preferred flavored drinks. As shown in Figure 6, BAHT, BAHM, BBHT, and BBHM presented higher average scores than the control drinks (BAN and BBN). On the other hand, tasters identified a difference between drinks with water A or B. Concerning CI, BA drinks were rated slightly higher, with a maximum value of 3.75 ± 0.89 in BAHT. These results agree with spectrophotometric colors measurements (Figure 3). In terms of aromatic intensity and quality, results showed significant differences between samples. BBHM (4.00 ± 0.76) was the most preferred, followed by BAHT (3.63 ± 1.19).

However, samples were slightly herbaceous without significant differences. Fruity and floral parameters showed significant differences; tasters described BA beverages as more floral (2.75 ± 1.04) and fruitier (2.88 ± 0.83) than BB beverages. Samples were seen as a little bitter and with a medium level of sweetness. Tasters identified the lowest acidity in BA beverages with an increase in both flavoring beverages (BAHT and BAHM) compared to the control. Turbidity was lowest in beverages with no significant differences in this parameter due to the filtering process through flavors extraction; the turbidity of grape juice used was <10. Finally, in global perception, tasters demonstrate a great preference for BAHT (3.63 ± 0.92) followed by BAHM (3.38 ± 0.92).

## 4 DISCUSSION

The samples prepared were subjected to physicochemical and sensory analysis to determine their different properties. Results demonstrated that samples had low pH values (Figure 2). The pH values differed slightly among the samples, although the same quantity of lemon juice was added during beverages preparation. The pH was lower for beverages prepared with low‐mineralized water (BA) compared to beverages prepared with high‐mineralized water (BB). The trend with total acids (Table 4) was the same: BA samples had higher levels of total acids (1.43 ± 0.06 g/L) compared with BB samples. This high acidity in sample drinks could be due to the low pH of the concentrate grape juice (pH = 3.5) used for the preparation of beverages, the presence of tartaric, malic, and citric acids in the grape juice composition (Cosme et al., 2018), and the use of lemon juice, which is characterized by its content of ascorbic acid (vitamin C) (Hooshyar et al., 2020) and citric acid (Gironés‐vilaplana et al., 2012). It should be noted that these values ensure the safety of the beverage by promoting resistance to microbial deterioration (Hani et al., 2019) including *Clostridium botulinium* (Porfírio et al., 2020). Furthermore, an acidic pH lower than 3.5 is important to obtain the required red color and the stability of anthocyanins (Hani et al., 2019). According to our results (Figure 3), the absorbance at 520 nm (an increase during 20 days) was higher in the BA sample where the acidity was higher (Figure 2). The same trend with CI and total phenolic content with higher values were marked in BA samples (Table 3). These results clearly show that the degree of mineralization of waters influenced the acidity of grape juice beverages and made a significant difference in the absorbance at 520 nm and color between the two groups of beverages. The color characteristic depends on the anthocyanins content, which are responsible for the red color of grape juice and present the most important indicator of grape juice quality (Burin et al., 2010; Dıblan & Özkan, 2021). The stability of anthocyanins is affected by different factors such as the chemical structure, pH, temperature, oxygen, concentration, light, enzymes, presence of co‐pigments, and food matrix composition (proteins, carbohydrates, ascorbic acids, minerals, salts, and sugars) (Morata et al., 2019; Ren & Giusti, 2021; Vidana Gamage et al., 2022) reported that the stability, CI, and absorption wavelength of anthocyanins depend on pH of the medium. In lower pH solutions, the predominant form of anthocyanins is the flavylium cation which shows an intense red color. In strong acidic media (pH 1–2), CI increases strongly. When pH increases, this form turns to uncolored carbinol pseudobase. The color becomes blue‐violet in a basic pH because of the transformation to a quinoidal base form. Consequently, anthocyanins are stable in low pH values. Additionally, the color change based on pH conditions is also influenced by the type of anthocyanins, cyanidin shows red color at pH < 3, violet color at pH 7 and 8, and blue color at pH > 11. However, peonidin has higher stability at high pH than other anthocyanidins; at acidic conditions, it shows red color to cherry, and at pH 8 shows a deep blue color (Chandra et al., 2021). Moreover, color stability also depends on the water concentration as the decrease in the concentration of water improves the deprotonation rate of the flavylium, lowering the stability of color (Chandra et al., 2021). Hydration reactions break the pyrylium ring aromaticity producing the loss of absorption properties and the transformation of the structure to an uncolored carbinol pseudobase that turn to an open chalcone shows a light‐yellow color (Morata et al., 2019).

Chandra et al. (2021) reviewed that cyanidin, pelargonidin, peonidin, and malvidin are the anthocyanidins responsible for the red color. Nevertheless, each red fruit has its own anthocyanin content, which makes its properties different from other fruits. Grapes and blueberry show the most diverse profile of anthocyanin pigments provided in the juices (malvidin, delphinidin, peonidin, cyanidin, and petunidin), while only two anthocyanidins are found in strawberry juice (cyanidin and pelargonidin).

According to Hooshyar et al. (2020), the glucoside forms of malvidin, delphinidin, cyanidin, pelargonidin, peonidin, and petunidin are the main abundant anthocyanins in red grapes. Our results (Table 5) present the same types of anthocyanins with different concentrations among samples and with a predominance of malvidin pigment in all samples, which is the main anthocyanin found in red grape juice among the six monomeric anthocyanins (Cosme et al., 2018). The concentration of anthocyanins in grape juices depends on cultivars, raw material, processing technology, and heat treatment (Cosme et al., 2018). Dıblan & Özkan (2021) mentioned that different concentration of monomeric anthocyanins was obtained from different cultivars, and these findings indicate that anthocyanins content of red grape juice depends on cultivar. An investigation of the effects of various clarification treatments on anthocyanins, color, phenolics, and antioxidant activity of red grape juice demonstrated that the clarification and the type of clarifying agents affect the anthocyanin content causing a reduction in monomeric anthocyanin content of red grape juice (Dıblan & Özkan, 2021). As well, polyphenols and anthocyanins are sensitive to heat and their degradation is dependent on temperature and can be easily broken during the heat treatment of fruit juices. Thus, a loss of food color during the processing can indicate anthocyanin degradation (Mirzaee et al., 2016). Ayoub et al. (2020) studied the influence of ohmic heating at different voltages on the different physicochemical properties of grape juice; results revealed a decrease in the anthocyanins content. Authors attributed the loss of anthocyanins to the instability of these pigments which led to their degradation through heating process.

**FIGURE 4:**
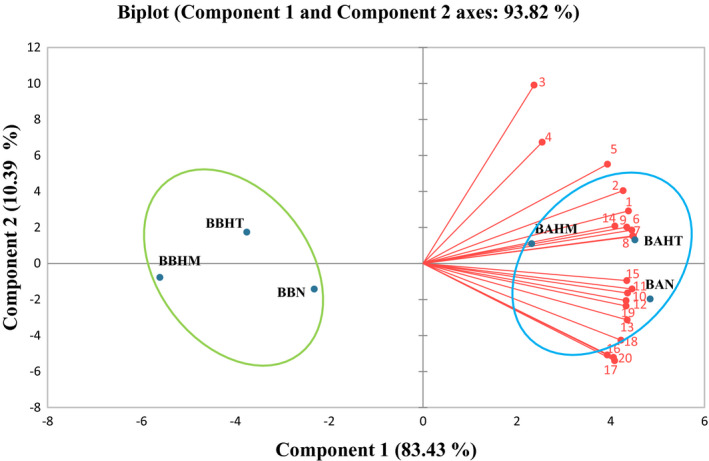
Principal component analysis (PCA) of color absorbance at 520 nm for 20 days

**FIGURE 5:**
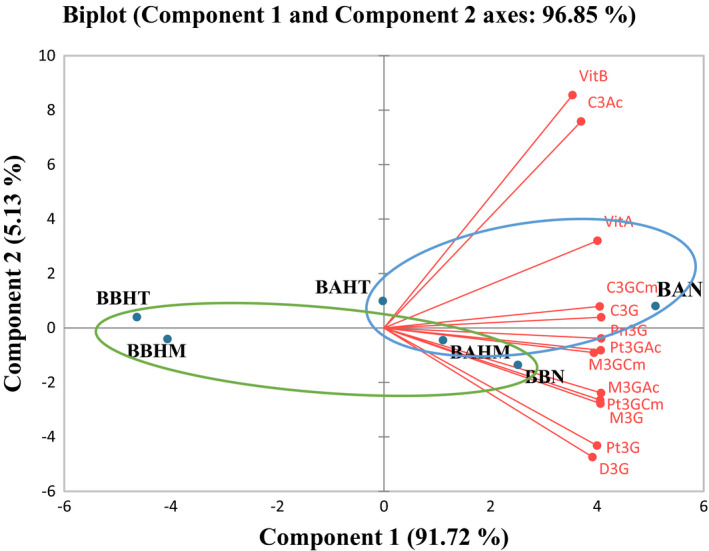
Principal component analysis (PCA) of anthocyanins determined in beverages by LC‐DAD

After performing PCA analysis on the color absorbance at 520 nm for 20 days, two principal components obtained explain 93.82% of the total variance: 83.43% of the variance was explained by the first component (PC1) and 10.39% of the variance by the second component (PC2). The samples are grouped into two distinct groups (Figure 4). BAN, BAHT, and BAHM samples belong to one group; BBN, BBHT, and BBHM samples are the second group. The first group represents beverages prepared with low mineral water and the second group represents those prepared with high mineral water. Concerning PCA analysis on the anthocyanin's parameter (Figure 5), two principal components obtained explain 96.85% of the total variance. The first component (PC1) explained 91.72% of the variance and the second component (PC2) explained 15.13% of the variance. Two groups were differentiated. One of them represents samples prepared with the low mineral water (BAN, BAHT, and BAHM) and the other includes samples prepared with the high mineral water (BBN, BBHT, and BBHM), with different distribution of BBN samples that did not follow the expected trend. Results of PCA analysis showed that already samples can be classified according to their mineral composition and their acidity, which influenced the anthocyanins and the color of the prepared beverages.

**FIGURE 6:**
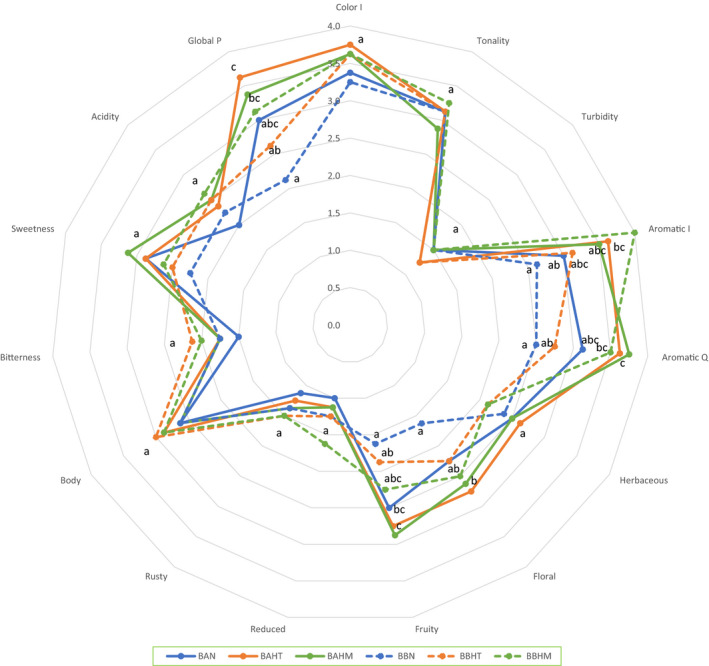
Sensory analysis of flavored beverages compared with the control beverages. The values are the averages from eight tasters. The same attributes with the same letter are not significantly different (*p* < .05)

Finally, results of the sensory analysis show that flavored beverages were preferred over control beverages (Figure 6). Panelists gave BAHT the highest mean score, including overall acceptance. This could be due to the preferred sour taste of the formulation, which was positively correlated with the highest level of total acid (1.43 ± 0.06 g/L) (Table 4) among beverages. Tasters greatly accepted the good taste and aroma of the mixture hop–tea (BAHT) added to grape juice, which was characterized by a strong aroma, flavor, and a pleasant taste due to the abundant content of vitamin C in grapes (Ayoub et al., 2020). In addition, color is an important sensory property when choosing a food product, especially in beverage products. In this case, grape juice was preferred as a source of natural colorant due to the high concentration of phenolic compounds that provide sensory characteristics (color, taste, and flavor), namely anthocyanins, which are responsible for the color of grape juice (Cosme et al., 2018). Attractive colors (red, orange, and purple) and water solubility of anthocyanins allow their incorporation into aqueous food systems as natural colorants (Morata et al., 2019). In addition, color is used by consumers to determine the quality of agricultural and food products because of the strong correlation between color and flavor. It has been noticed that the identification of flavor decreases when the colors of food products are different from the expectations of consumers (Chandra et al., 2021). On the other hand, besides their pleasant flavors and aroma, spices have helpful effects on human health and act as natural preservatives (Ivanišová et al., 2005; Potortì et al., 2019; Souza et al., 2020). According to Moghaddam et al. (2018), fruits, vegetables, and herbs beverages are considered as health‐promoting agents because of their content on bioactive compounds including phenolic compounds, antioxidant agents, and organic acids. This encourages the combination of good sensory properties (color and pleasant aroma) from natural sources and health benefits because of contents rich in phenolic compounds of grape juices and herbs in one product, which could be acceptable to consumers increasingly searching for products free of artificial additives.

## 5 CONCLUSION

In this study, new natural beverages were formulated from a combination of concentrated grape juice with two different mineral waters (Bezoya and Solan de Cabras), using lemon juice for pH correction, naturally flavored with herbs and spices extracts. The results of color measurement and pH indicated that beverages prepared with low mineral water (BA) present high acidity and high CI. On the other hand, the most interesting in terms of sensory analysis is the beverage prepared with low mineral water and flavored with a mixture of hop and tea (BAHT). The most abundant anthocyanin in prepared drinks was malvidin‐3‐O‐glucoside, followed by peonidin‐3‐glucoside. Moreover, grape juice, lemon juice, and plant extracts have great potential in the development of a healthy fruit drink due to the antioxidant activity provided by their polyphenolic compounds. This is presented as an alternative for consumers looking for drinks that are less artificial and more beneficial to their health. Additionally, the beverages prepared contribute to sustainable development as they are produced under organic production conditions.

## ACKNOWLEDGEMENTS

The authors acknowledge the Algerian government for the scholarship offered to Yasmina Bendaali giving an opportunity to carry out this project. Authors also thank Fernando Boned and Fernando Chivite (in memoriam) for their full support in this project.

## CONFLICT OF INTEREST

The authors declare that they have no conflict of interest.

## COMPLIANCE WITH ETHICS REQUIREMENTS

This study does not contain any studies with human or animal subjects.

## Data Availability

Supplementary data are available upon reasonable request.
